# CaO-B_2_O_3_-SiO_2_ glass fibers for wound healing

**DOI:** 10.1007/s10856-021-06618-3

**Published:** 2022-01-24

**Authors:** Seiji Yamaguchi, Tamaki Takeuchi, Morihiro Ito, Tadashi Kokubo

**Affiliations:** Department of Biomedical Sciences, College of Life and Health Sciences, Chubu University, 1200 Matsumoto-cho, Kasugai, Aichi 487-8501 Japan

## Abstract

It was reported by Jung and Day in 2011 that a cotton-like glass fiber pad made of borate glass 13-93B3 demonstrated a remarkable wound healing effect. It was approved for sale as a novel wound dressing in the management of acute and chronic wounds in 2016. However, the detailed mechanism of its wound healing effect has not been reported. In the present study, glass fibers of different composition in the system CaO-B_2_O_3_-SiO_2_ were prepared and their in vitro properties investigated to determine the role of the constituent components in wound healing. Fine glass fibers that were 0.6–2.0 μm in diameter were obtained by a melt blown method. However, these fibers were accompanied by small glass beads because of the low viscosity of the glass melts. 13-93B3 glass released an appreciable amount of borate and calcium ions into simulated body fluid (SBF). The amounts of these released ions decreased with partial replacement of the B_2_O_3_ in 13-93B3 with SiO_2_. The addition of large amounts of the borate and calcium ions into the culture medium decreased the viability of the L929 fibroblasts. Partial replacement of the B_2_O_3_ in 13-93B3 with SiO_2_ induced the formation of an apatite-like phase amenable to the adsorption of biological components on its surface in SBF. The wound healing effect of these glass fibers of different composition is worth examining in future animal experiments.

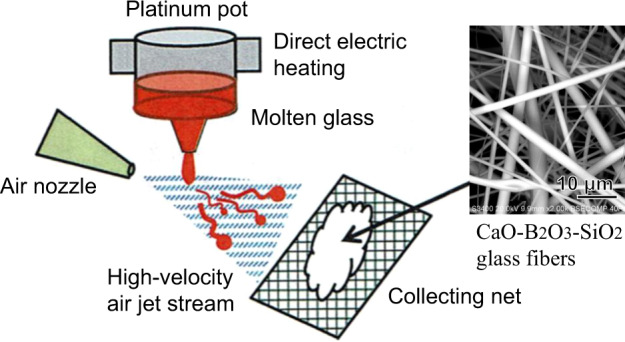

## Introduction

Certain Na_2_O-CaO-SiO_2_-P_2_O_5_ glasses were found to bond to living bone by Hench et al. in 1970 and named Bioglass^®^ [1]. Subsequently, various kinds of bone-bonding glasses and glass-ceramics have been derived from them. Some of these have been successfully subjected to clinical applications as bone-repair materials [2].

It was also found by Wilson in 1981 that Bioglass^®^ can even bond to soft living tissues [3]. However, this characteristic of Bioglass^®^ has not been used clinically for a long period of time. In 2011 Jung and Day demonstrated that a cotton-like glass fiber pad of borate glass named 13-93B3 that is composed of K_2_O, CaO and B_2_O_3_ exhibited a remarkable wound healing effect [4–6]. These glass fibers received USA FDA approval as a novel wound dressing in the management of acute and chronic wounds in 2016 [7]. Great potential of bioactive glasses in soft tissues repair was recently disscussed by Bino et al. [8, 9].

However, systematic study of specific mechanism of action in accelerating the wound healing processes has not been reported [10]. Zhou et al. reported that the borate 13-93B3 glass fibers display faster wound healing than the conventional silicate Bioglass 45S5 glass fibers and attributed the faster healing by the former glass to the boron component in the glass [11]. On the other hand, Liu et al. attributed the faster healing by the former to a faster release of calcium ions from the glass [12].

Lin et al. [13], Zhao et al. [14], and Chen et al. [15] showed that the addition of copper and/or zinc ions to the borate glass promotes its healing effect. Recently, Thyparambil et al. reported that 13-93B3 glass can trigger phenotypic change in adipose stem cells [16]. Nevertheless, the detailed mechanism of the wound healing effect of the 13-93B3 fibers remains to be elucidated.

In the present study, glass fibers of different composition in the system CaO-B_2_O_3_-SiO_2_ were prepared and their in vitro properties investigated to identify the specific role of the constituent components of the glasses in wound healing.

## Materials and methods

### Preparation of the glasses

**Fig. 1:**
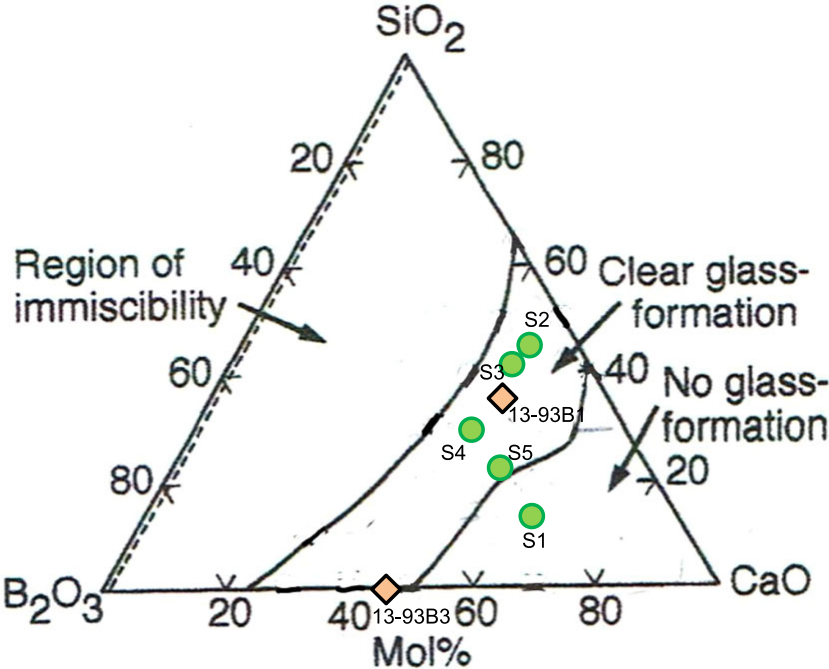
Compositions of examined glasses, which were plotted in the compositional triangle CaO-B_2_O_3_-SiO_2_

Batch mixtures of ~300 g yielding the various oxide compositions given in Table 1 were prepared from optical silica sand and reagent grade oxides and carbonates. 13-93B3 in Table 1 is a composition of the borate glass fibers which was reported by Jung et al. to be effective for wound healing [4–6], while 13-93B1 is composition of a silicate glass in which the B_2_O_3_ of 13-93B3 is partially replaced with SiO_2_ [17]. S1 to S5 are compositions of silicate glasses in which the relative contents of the constituent components of 13-93B1 are varied. The glass-forming region in the system CaO-B_2_O_3-_SiO_2_ was determined by Ohtsuki et al. [18] by the method of quenching 30 g melted onto a steel plate. If the compositions given in Table 1 are plotted on the compositional triangle of CaO-B_2_O_3_-SiO_2,_ Fig. 1 is obtained, in which components other than B_2_O_3_ and SiO_2_ are included in the CaO. It can be seen from Fig. 1 that the compositions of the examined glasses are distributed in a wide range, almost in the glass-forming region.

**Table 1:** Compositions of examined glasses

	Composition/ mol %						
Sample	Na_2_O	K_2_O	MgO	CaO	SiO_2_	B_2_O_3_	P_2_O_5_
S1	5.7	7.5	7.4	41.0	15.8	22.0	0.6
S2	5.1	6.8	6.6	27.5	45.6	7.0	1.4
S3	5.0	9.0	7.0	22.1	41.8	13.2	1.9
S4	5.0	9.0	7.0	22.1	29.8	25.2	1.9
S5	5.0	9.0	7.0	35.0	21.1	21.0	1.9
13-93B1	6.0	7.9	7.7	22.1	36.4	18.2	1.7
13-93B3	6.0	7.9	7.7	22.1		54.6	1.7

**Fig. 2:**
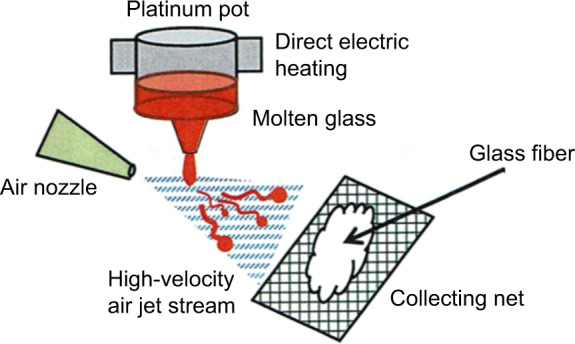
Schematic representation of preparation of glass fibers

The batch mixtures of these compositions were put into a 500 ml Pt10Rh crucible and melted in a SiC furnace at 1200–1550 °C for 4 h. The melts were poured on to a carbon plate and pressed into a plate about 8 mm thick. The obtained glass plate was crushed into small grains and put into an electrically heated platinum pot 90 ml in volume to be re-melted, as shown in Fig. 2. The molten glass was drawn through a narrow nozzle at the bottom of the pot and blown with a high-velocity air jet stream at a speed of 400 m/s under a pressure of 500KPa. The short fine fibers that formed were trapped on a steel net.

Glass grains 300–500 μm in size were prepared from the glass plates to measure the ion release into a simulated body fluid (SBF).

Rectangular specimens 25 × 35 × 0.8 mm^3^ in size were prepared from the glass plate to analyze changes in the surface structure of the glasses in SBF.

### The morphology and structure of the glass fibers

The as-prepared glass fibers were observed under scanning electron microscopy (SEM: s-3400N type, Hitachi Co. Tokyo, Japan) with a voltage of 20 kV. The diameters of fifty glass fibers were measured and averaged.

Structure of the as-prepared glass fibers were analyzed by powder X-ray diffraction (RINT2000, Rigaku, co., Japan) using CuKα X-ray at 40 kV and 40 mA.

### Measurement of the liquidus temperature of the glasses

Small glass particles were put on a platinum boat and placed in a temperature-gradient furnace. After 16 h. the boat was taken out from the furnace and the highest temperature at which precipitation of the crystal was observed was taken as the liquidus temperature of the glass.

### Measurement of the viscosity of the glass melts and the density of the glasses

The viscosity of the glass melts just above the liquidus temperature was measured by a ball-pulling up method.

The density of the prepared glasses was measured by an Archimedean method.

### Measurement of the transition temperatures of the glasses

As-prepared glass fibers were pulverized and subjected to TG-DTA (Thermo plus TG8120, Rigaku, Co., Japan) analyses to measure the glass transition temperatures and crystallization temperatures.

### Analysis of surface structural changes of the glasses in SBF

In order to investigate the reaction of the examined glasses with body fluid, change in the surface structure of the glasses in a SBF was studied by the following method.

Glass pates 25 × 35 × 08 mm^3^ in size were soaked in 100 ml of a SBF in which the ion concentrations (Na^+^ 142.0, K^+^ 5.0, Ca^2+^ 2.5, Mg^2+^ 1.5, Cl^–^ 147.8, HCO^3–^ 4.2, HPO_4_^2–^ 1.0, and SO_4_^2–^ 0.5 mM) were nearly equal to those of human blood plasma at 36.5 °C, and the area of the glass plate contact with SBF was maintained at 13 mm^2^/ml. The SBF was prepared by dissolving reagent grade NaCl, NaHCO_3,_ KCl, K_2_HPO_4_•3H_2_O, MgCl_2_•6H_2_O, CaCl_2_, and Na_2_SO_4_ (FUJIFILM Wako Chemicals, Japan) in ultrapure water, and then buffered at pH = 7.4 with tris(hydroxymethyl)aminomethane (CH_2_OH)_3_CNH_2_ (FUJIFILM Wako Chemicals, Japan) and 1 M HCl (Kanto Chemical Co., Inc. Japan) at 36.5 °C [19]. After soaking in the SBF for 1 week, the samples were gently removed from the SBF and dried in air without washing to prevent any peeling off of the surface layers from the glass substrates. Apatite formation on their surfaces was examined by thin-film X-ray diffraction (SmartLab, Rigaku Co., Japan).

### Measurement of ion release from the glasses into SBF

The 0.6–0.7 g of glass particles were 300–500 μm in diameter, which corresponds to the relative density of the glass × 0.256. They were used to measure the ion release from the glasses into SBF, with a constant surface area exposed to the SBF. The glass particles were soaked in 60 ml of SBF at 36.5 °C for 2 days. During the soaking, the SBF was manually mixed once after 1 day. After the soaking, the SBF was passed through a filter and the concentrations of B, Ca and Si in the filtered SBF were measured by inductively coupled plasma emission spectroscopy (ICP, SPS-3520UV, Hitachi High-Tech Science Corporation, Japan).

### Cell study

#### Effect of the borate and calcium ion concentrations on cell viability

The effects of pH and the concentrations of borate and calcium ions on cytotoxicity were examined using L929 fibroblast cells by a serial dilution method. The cells were pre-cultured on a 96 well plate so as to reach confluency in 100 μl of DMEM along with 5% fetal bovine serum (FBS) and 1% penicillin/streptomycin at 37 °C in a 5% CO_2_ atmosphere. Then the cells were cultured for another 3 days after the medium was replaced with a fresh one with the pH adjusted to 7.83–9.94 by the addition of Ca(OH)_2_ (Reagent grade; Kanto Chemical Co., Inc., Tokyo, Japan). It should be noted that the pH of the medium was initially 7.83 and this was increased effectively by the reagent Ca(OH)_2_ with only a negligible increase of calcium ions in the medium. The cells were also cultured in the medium with concentrations of borate (0 – 688.7 mM) or calcium ions (2.1 – 991.6 mM) by the addition of H_3_BO_3_ (Reagent grade; Kanto Chemical Co., Inc., Tokyo, Japan) and CaCl_2_ (Reagent grade;Nacalai Tesque Inc., Kyoto, Japan), respectively. After the culture, 10 μl of the cell count reagent WST-8 (Dojindo Laboratories Co., Ltd., Kumamoto, Japan) was added to each well, then the well plate was shaken with a plate shaker for 1 min and stored in an incubator at 37 °C in 5% CO_2_ atmosphere for 2 h. The absorbance at 450 nm, which is attributed to the formazan product derived from living cells, was quantified by a Microplate reader (Biotrack II, Amersham Biosciences Corp, USA). In order to determine the effects of low concentrations of the borate and calcium ions on cell viability, L929 cells were separately cultured using the medium with 5, 25, 50, and 100 mM borate or 4 and 6 mM calcium ions. The medium was used without any additives as a control. The experiment was repeated three times and statistically analyzed with Dunnett’s test to compare control against the treated groups in which the cells were cultured in the medium with the additives of H_3_BO_3_ and CaCl_2_. The significance was accepted at *P* < 0.05 while single and double asterisks show *P* < 0.01 and *P* < 0.001, respectively.

#### Effect of extracted ions from glass fibers on cell viability

Seventy mg of glass fibers were immersed in 7 ml of medium through a 0.22 μm membrane filter and remained for 3 days at 36.5 °C, which allowed transportation of the ions dissolved in the medium inside the filter to the medium outside the filter. The same amount of hydrocolloid (DuoACTIVE ET, ConvaTec Japan), which is one of the popular wound dressing materials that do not release any ions, was used as a control.

After the soaking, the medium with the ions extracted from the glass fibers was subjected to two serial dilutions and used for the cell viability test in the same manner as described in section 2.7.1.

## Results

### Morphology and structure of the glass fibers

**Fig. 3:**
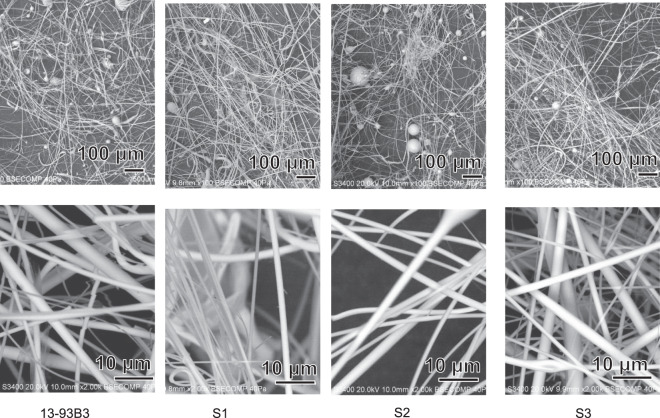
SEM pictures of as-prepared glass fibers

Figure 3 shows the SEM images of the glass fibers prepared by the method described in Section 2.1. It can be seen from Fig. 3 that all of the glass fibers obtained displayed smooth surfaces, but these were accompanied by small glass beads up to several dozen μm in diameter. Their relative amount was a little larger for 13-93B3 than for the other compositions. The diameter of the glass fibers ranged from 0.2 to 3.0 μm, mainly located in the range of 0.6 to 2.0 μm. Their average diameters are given in Table 2.

**Table 2:** Some physical properties of examined glasses

Sample	Diameter/μm	Liquidus temperature/ C	Viscosity /dPa-s	Density/g∙cm^-3^	Glass transition temperatures/°C
S1	0.6 ± 0.4	1010	10^0.4^	2.7033	500
S2	1.0 ± 0.7	1105	10^1.7^	2.6773	597
S3	1.0 ± 0.8	1100	10^1.3^	2.6196	582
S4	0.9 ± 0.7	1105	10^0.6^	2.5973	558
S5	0.9 ± 0.7	940	10^0.5^	2.6673	525
13-93B1	0.9 ± 0.7	1045	10^1.3^	2.6206	563
13-93B3	1.1 ± 0.8	1000	10^0.4^	2.5133	542

**Fig. 4:**
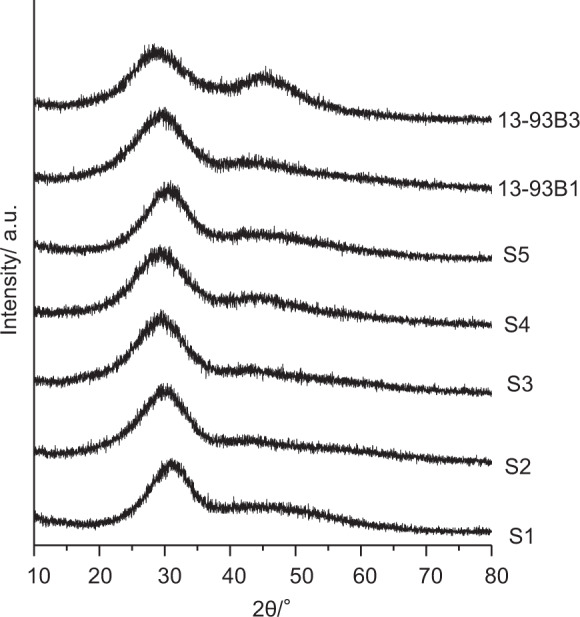
Powder XRD patterns of glasses fibers

Figure 4 shows powder X-ray diffraction patterns of the glass fibers. It can be seen that all the glass fibers showed broad hallo at around 30 and 45° in 2θ, indicating that both the fibers and beads consist of glassy phase.

### Some physical properties of the glasses

The liquidus temperature of the glasses, viscosity of the glasses just above the liquidus temperature, and density of the glasses, which were measured by the methods described in Sections 2.3 and 2.4, are given in Table 2. The liquidus temperature of the glasses was in a range from 940 to 1105 °C. The viscosity of the glasses just above the liquidus temperature was in the range of 10^0.4^ to 10^1.7^ dPa^-s^. This viscosity is much lower than that of common commercial glass fibers. Therefore, conventional methods for producing glass fibers cannot be applied to the present glasses.

**Fig. 5:**
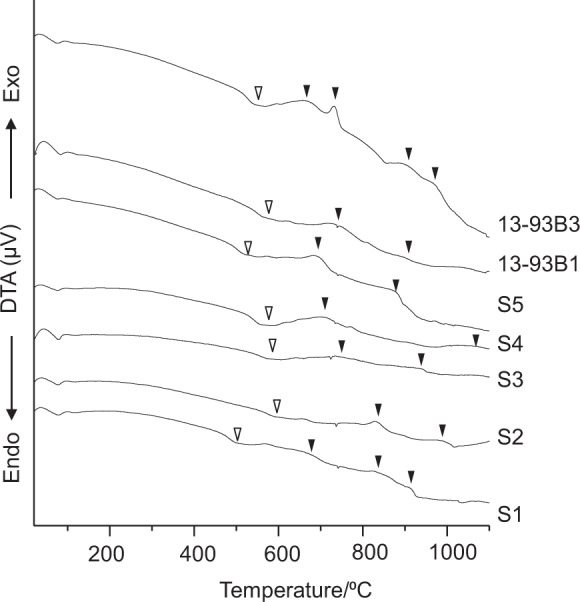
DTA profiles of glass fibers. ∇ Glass transition temperature ▼Crystallization temperature

Figure 5 shows DTA profiles of the glass fibers. The endothermic peaks and exothermic peaks on the profiles are attributed to the glass transition temperatures and crystallization temperatures of each glass fibers, respectively. They are summarized in Table 2. It can be seen from Table 2 that the glass transition temperatures are located in the range 500–597 °C.

### Surface structural changes of the glasses in SBF

**Fig. 6:**
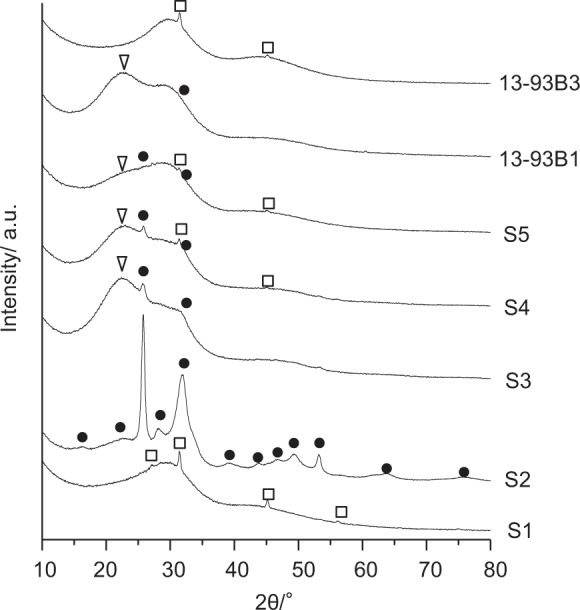
Thin-film XRD patterns of the surfaces of the glasses after soaking in SBF for 1 week. • Apatite, ∇ Silica gel, □ NaCl

The thin-film X-ray diffraction patterns on the surfaces of the examined glasses after having been soaked in SBF for 1 week are shown in Fig. 6. S2 gave sharp diffraction peaks ascribed to crystalline apatite at 26 ° and 32 ° in 2 θ. Broad diffraction peaks around these angles were observed also for S3, 4, 5 and 13-93B1. This indicates that S2, 3, 4, 5 and 13-93B1 formed an apatite or apatite-like phase on its surface in SBF. S3, 4, 5 and 13-93B1 gave another broad diffraction peak around 24 ° in 2 θ. This diffraction peak might be attributed to a silica gel formed on their surfaces, as reported for 45S5 by Liu et al. [12]. S1 and 13-93B3 gave only small diffraction peaks that were ascribed to the NaCl precipitated from SBF, in addition to a broad peak ascribed to the original glass, even after soaking in SBF. These results are summarized in Table 3.

**Table 3:** Surface change of glasses and ion concentrations in SBF

		Ion concentration in SBF after 2d/mM		
Sample	Surface phase formed in SBF	B	Ca	Si
S1	No	14.5	12.6	0.3
S2	Apatite	1.9	4.8	1.4
S3	Apatite-like phase + Silica gel	4.1	4.4	1.6
S4	Apatite-like phase + Silica gel	6.4	4.0	1.0
S5	Apatite-like phase + Silica gel	6.7	5.5	0.8
13-93B1	Apatite-like phase	5.2	4.2	1.3
13-93B3	No	61.1	10.4	0

### Ion release from the glasses into SBF

The pH of the SBF that was initially 7.4 increased to 8.0–8.3 after soaking of the glasses for 2 days, independent of the glass composition.

Table 3 shows the B, Ca and Si concentrations in SBF after the glasses were soaked for 2 days. It can be seen in Table 2 that 13-93B3 released large amounts of the borate and calcium ions into SBF. It should be noted here that SBF contained 2.5 mM calcium ions even before the soaking of the glass. Its concentration increased to up to 10.4 mM after the soaking of the glass. Other glasses released smaller amounts of the borate and calcium ions, except S1 which released fairly large amounts of borate and calcium ions among the silicate glasses. All the silicate glasses released a small amount of silicate ions.

### Cytotoxicity of the ions released from the glasses

**Fig. 7:**
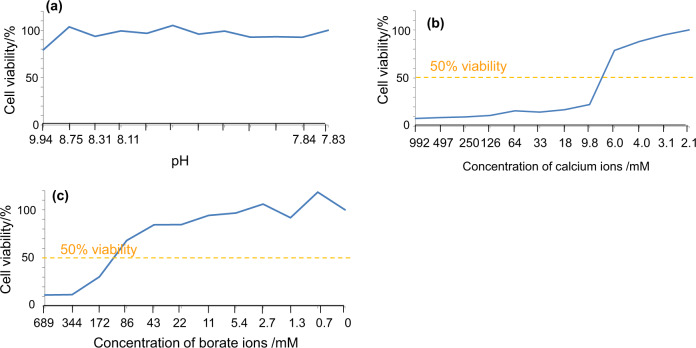
Effect of (**a**) pH and concentrations of (**b**) calcium, and (**c**) borate ions in the medium on cell viability of L929 cells

Figure 7 shows the effects of the pH and concentrations of borate and calcium ions on the viability of L929 fibroblast cells by a serial dilution method. No apparent decrease of cell viability was observed in the pH range of 7.83–9.94. In contrast, cell viability significantly decreased to lower than 50% when the concentrations of calcium and borate ions in the medium became higher than 9.8 and 86.1 mM, respectively.

**Fig. 8:**
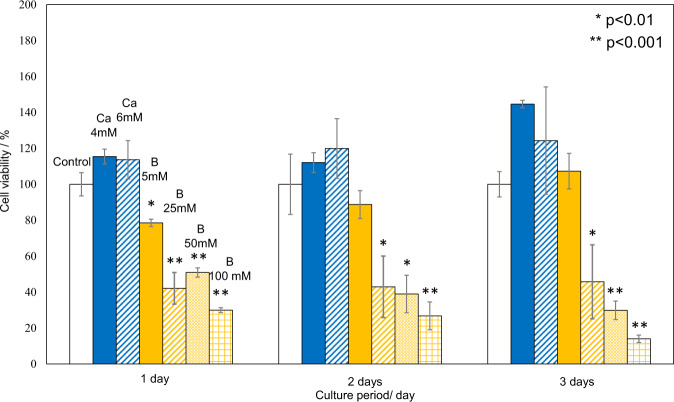
Effect of low amount of calcium and borate ions on cell viability of L929 cells. *, **: statistical significant difference against control

The effects of these ions on cell viability were further investigated using medium containing various amounts of borate and calcium ions lower than the critical values at which cell viability decreased to less than 50%. The results are shown in Fig. 8. It can be seen from Fig. 6 that a tendency toward increased cell viability observed for 6 mM CaCl_2_ on 1 day, and 4 and 6 mM CaCl_2_ on day 3, although statistical analysis did not detect a significant increase. In contrast, borate ions equal to or higher than 25 mM significantly decreased cell viability throughput the culture periods.

**Fig. 9:**
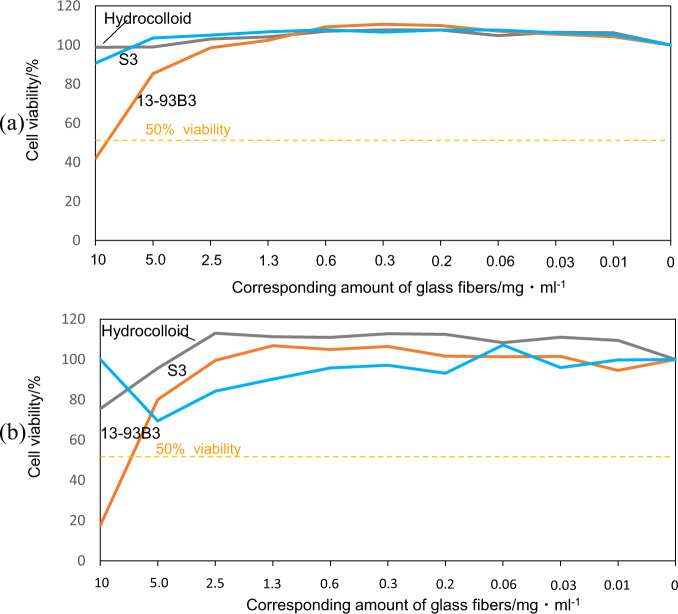
Effect of released ions extracted from 13-93B3 and S3 glass fibers on cell viability of L929 cells after culture periods of (**a**) 1 and (**b**) 3 days

Figure 9 shows the cell viability after culture for 1 and 3 days in the medium immersed with various amounts of 13-93B3 and S3 glass fibers for 3 days. It can be seen from Fig. 9 that the cell validity decreased to less than 50% within 1 day for 10 mg/ml of 13-93B3 glass fibers, while it maintained a value greater than 50% for up to 3 days for S3 glass fibers.

The addition of hydrocolloid into the medium had no effect on the cell viability.

## Discussion

According to Jung and Day [4–6], it is important for the glasses for wound healing to take the form of cotton-like pad of fine fibers, since fine fibers can easily release the ions that are effective for wound healing because of their high specific surface area, and a cotton-like pad can be easily fitted to any shape of wound and provide many void spaces for maintaining a moist environment in which various kinds of biological components effective for wound healing are contained, such as vascular endothelial growth factor, basic fibroblast growth factor and vascular cell adhesion protein. However, the preparation of fine glass fibers from melts with a low viscosity, as shown in Table 2, is difficult by conventional methods. Therefore, the special method given in Fig. 2 was used in the present study. As the result, fine glass fibers 0.6 to 2.0 μm in diameter were successfully obtained. However, all the obtained glass fibers were accompanied by small glass beads, as shown in Fig. 3, even using this method. These glass beads are not desirable, since they tend not only to disturb uniform ion release from the glass fibers, but also to induce cicatricial tissue formation. The amount of these glass beads was smaller for silicate glasses than the borate glass 13-93B as seen from Fig. 3, since the silicate glasses give higher viscosities (see Table 2).

**Fig. 10:**
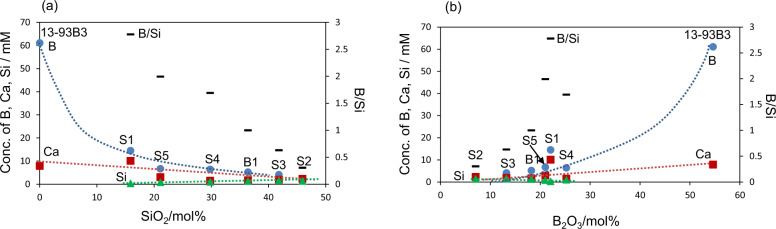
Concentrations of calcium, borate and silicate ions released from the glasses into SBF for 2 days as a function of (**a**) SiO_2_ and (**b**) B_2_O_3_ contents of glasses

It is apparent from Table 3 that the borate glass 13-93B releases appreciable amounts of borate ions as well as calcium ions into SBF, while these ion releases are considerably decreased by partial replacement of the B_2_O_3_ in the glass with SiO_2,_ However, even silicate glass releases fairly large amounts of borate and calcium ions, depending on the specific composition (see S1). The concentrations of calcium, borate and silicate ions released from the glasses into SBF over 2 days, which are given in Table 3, were plotted against B_2_O_3_ and SiO_2_ contents of the glasses in Fig. 10. The amount of calcium ions released was calculated by subtracting 2.5 mM, which is the original concentration of calcium ions in the medium, from the concentration of calcium ions in SBF after the ion release test. It can be seen from Fig. 10 that the release of calcium ions from the glasses increases with an increasing B_2_O_3_ content of the glasses, while it decreases with increasing SiO_2_ content. The release of borate ions from the glasses increases with an increasing B_2_O_3_ content of the glasses, while it decreases with increasing SiO_2_ content. The release of Si from the glasses decreases with an increasing B_2_O_3_ content of the glasses, while it increases with increasing SiO_2_ content. These results show that the amounts of calcium, borate and silicate ions released from the glasses into body environment can be controlled by the CaO, B_2_O_3_ and SiO_2_ contents of the glasses.

It is speculated that not only the ions released from the glasses, but also the composition and structure of the surface layer of the glass fibers after being exposed to body fluid might exert a powerful influence on the wound healing process, since the apatite or apatite-like layer formed on the surface might adsorb various kinds of important biological components that are able to induce wound healing. Figure 6 and Table 3 show that S2, S3, S4, S5 and 13-93B1 form the apatite or apatite-like layer on their surfaces in SBF within 1 week. The formation of the apatite or apatite-like layer is hypothetically induced by a silica-rich layer formed on their surfaces in SBF. It has been reported that silica-containing glasses generally form a silica-rich layer on their surfaces to induce apatite formation in SBF [20]. For S3, 4, 5 and 13-93B1, the formation of the silica gel layer was confirmed, as shown in Fig. 6.

Soaking of the present glasses in SBF increased the pH of SBF to 8.0–8.3, as described in Section 3.4. As may be seen in Fig. 7, the increase of pH in this range for the culture medium has little effect on the viability of L929 fibroblast cells. On the other hand, as shown in the same figure, the addition of calcium and borate ions to the culture medium in amounts larger than 9.8 and 86.1 mM, respectively decreased the viability of L929 fibroblast cells to levels lower than 50%. Figure 8 shows that the addition of 4–6 mM calcium ions increases the cell viability, while the addition of 25–100 mM borate ions largely decreases the cell viability.

It has also been reported that 3 mM of the calcium ions in the medium increased proliferation and the mRNA levels of BMP-2 and 4 human gingiva-derived fibroblasts [21], but exposure to calcium ions equal to or more than 100 mM for 5 min decreased the viability of Schwann cell to less than 50% [22]. On the other hand, it has been reported that the viability of human tooth stem cells was significantly decreased, to 80%, by 20 mM borate ions, and was further decreased, to 60%, with an increase of the borate ion concentration to 70 mM [23].

On the basis of these findings, it is speculated that the calcium ions released from silicate S2, S3, S4, S5, and 13-93B1 glasses in an amount less than 6 mM (See Table 2) exerts a positive effect on wound healing, while the borate ions released from the borate 13-93B3 glass in an amount of ~60 mM exerts a negative effect on wound healing.

These results and speculative account are consistent with the results of tests of the viability of L929 cells cultured in the medium where the glass fibers were immersed, give in Fig. 9. The cell viability decreased to a level lower than 50% when the cells were cultured in the medium in which 13-93B3 glass fibers larger than 5.0 mg/ml were immersed, whereas it was not affected by S3 silicate glass.

The effect of these glass fibers on wound healing will be investigated in animal experiments in a subsequent study.

## Summary

Fine glass fibers 0.6 to 2.0 μm in diameter of various composition in the system Na_2_O-K_2_O-MgO-CaO-B_2_O_3_-SiO_2_-P_2_O_5_ were prepared by a melt blown method. The melts of these various compositions were of a low viscosity above the liquidus temperature, and hence the glass fibers could not be formed from their melts without being accompanied by small glass beads. It was found that partial replacement of the B_2_O_3_ with SiO_2_ in the borate glass 13-93B3, which is known to be a wound-healing glass, decreased the amount of the glass beads.

When the glasses were soaked in SBF, 13-93B3 glass released an appreciable amount of borate and calcium ions into SBF while partial replacement of the B_2_O_3_ with SiO_2_ in the 13-93B3 glass considerably decreased the amount of the borate and calcium ions that were released. The silicate glasses derived from 13-93B3 glass by partial replacement of the B_2_O_3_ with SiO_2_ tended to form an apatite or apatite-like phase on their surfaces in SBF.

It was also shown that the addition of large amounts of borate and calcium ions to the culture medium decreased the viability of L929 fibroblast cells. The effect of a cotton-like pad of these glass fibers on wound healing will be examined in animal experiments.
